# Supplementary material for: Protein Disulfide Isomerase 4 Is an Essential Regulator of Endothelial Function and Survival
Source: Int J Mol Sci. 2024 Mar 31;25(7):3913. doi: 10.3390/ijms25073913 (PMC11011381; doi:10.3390/ijms25073913)
Supplement: Supplementary file 1 [file ijms-25-03913-s001.zip › ijms-2815517-supplementary.pdf]

Supplementary Figure S1

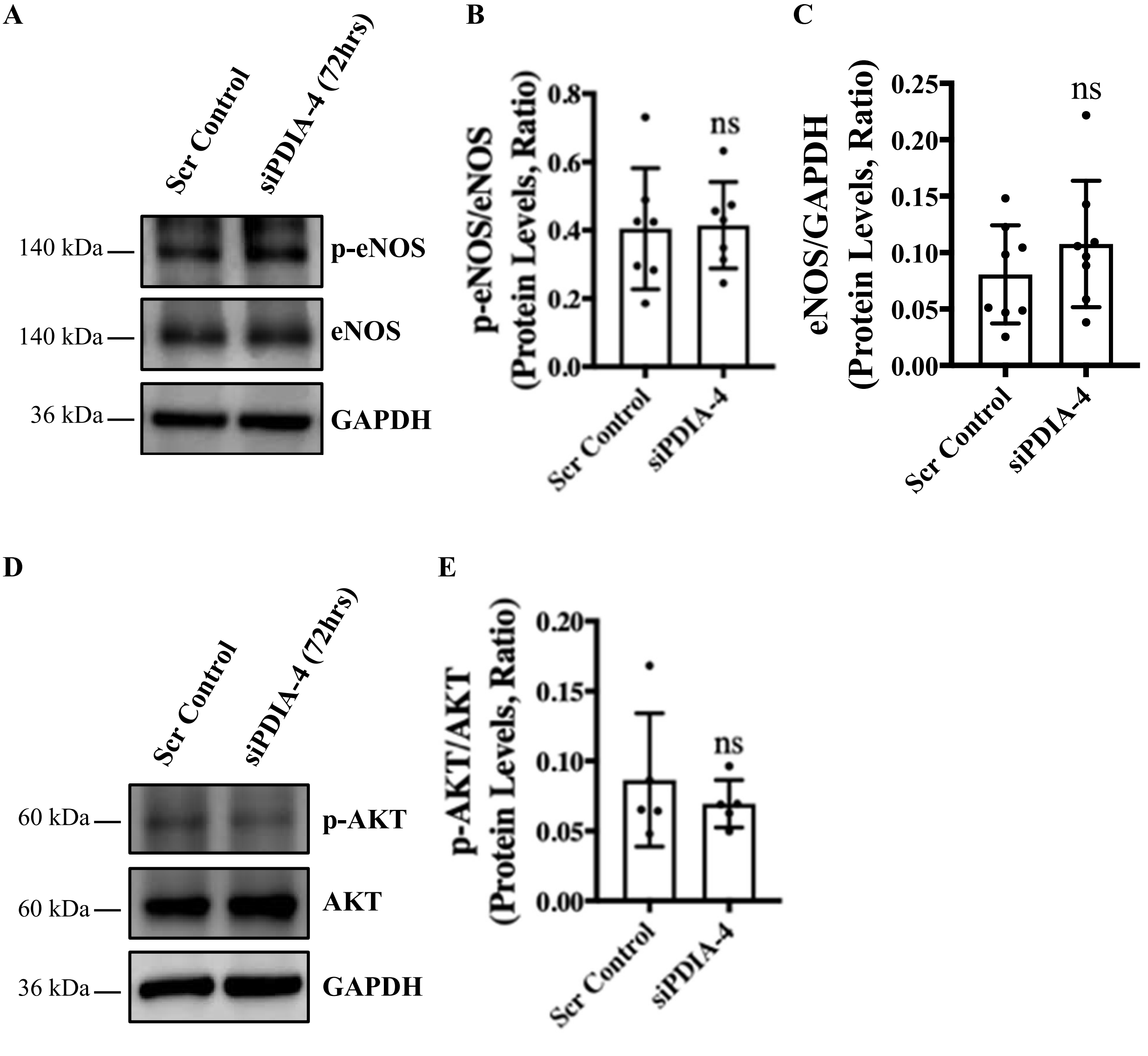

Supplementary Figure S1. HUVECs were reverse transfected with si*PDIA-4* or scrambled control and incubated in complete EGM-2 medium for 72hrs. Proteins were extracted and western blot was performed targeting eNOS, p-eNOS or GAPDH **(A)**, AKT, p-AKT or GAPDH **(B)**, and bands were quantified **(B, C, E)** (n=5-7). ns=non-significant.

Supplementary Table S1

Table S1. List of primers used to amplify genes

**Primer Name**	**Sequences (5’-3’)**
Hsa-*PDIA-4-F*	5'-CACGCTTGTGTTGACCAAAGA-3'
Hsa-*PDIA-4*-R	5'-AATTGGAGGAGAACGCTTGCT-3’
Hsa-*TGFB1-F*	5'-TACAGCACGGTATGCAAGCC-3'
Hsa-*TGFB1-R*	5’-GCAACCGATCTAGCTCACAGAG-3’
Hsa-*SMAD3-F*	5 '-TGGACGCAGGTTCTCCAAAC-3'
Hsa-*SMAD3-R*	5 '-CCGGCTC GCAGTAGGAAC-3'
Hsa-*SMAD4-F*	5 '-CTCATGTGATCTATGCCCGTC-3'
Hsa-*SMAD4-R*	5'-AGGTGATACAACTCGTTCGTAGT-3'
Hsa-*CD31-F*	5'-TCCTGAGGGTCAAGGTAATAGC-3’
Hsa-*CD31-R*	5'-CTCCAGACTGTACATCGTTACCC-3'
Hsa-*VE-Cadherin-F*	5 '-GAGTCTTCAGCTCACGGACA-3'
Hsa-*VE-Cadherin-R*	5 '-CAGCTCTGTGAGCCTCTGC-3'
Hsa-*SLUG-F*	5 '-ATCTGCGCiCAAGGCGTTTTCCA-3'
Hsa-*SLUG-R*	5 '-GAGCCCTCAGATTTGACCTGTC-3'
Hsa-*TGFBR1-F*	5'-TACCTGAACCCGTGTTGCTCTC-3'
Hsa-*TGFBR1-R*	5'-GTTGCTGAGGTATCGCCAGGAA-3'
Hsa-*TGFBR2-F*	5 '-GTCTGTGGATGACCTGGCTAAC-3'
Hsa-*TGFBR2-R*	5 '-GACATCGGTCTGCTTGAAGGAC-3'
Hsa-*FSP1-F*	5 '-TCTTGGTCTGGTCTCAACGG-5'
Hsa-*FSP1-R*	5 '-TGTCACCCTCTTTGCCTGAG-3'
Hsa-*N-Cadherin-F*	5 '-CCTCCAGAGTTTACTGCCATGAC-3'
Hsa-*N-Cadherin-R*	5 '-GTAGGATCTCCGCCACTGATTC-3'
